# Editorial: Bone cell differentiation in health and disease

**DOI:** 10.3389/fendo.2022.1115444

**Published:** 2022-12-16

**Authors:** Michaela Tencerova, Chandi C. Mandal

**Affiliations:** ^1^ Laboratory of Molecular Physiology of Bone, Institute of Physiology of the Czech Academy of Sciences, Prague, Czechia; ^2^ Department of Biochemistry, School of Life Sciences, Central University of Rajasthan, Ajmer, India

**Keywords:** osteoblast differentiation, osteoclast differentiation, metabolic and rare bone disease, inflammatory disease, molecular mechanism

Bone is a dynamic organ that undergoes remodeling processes throughout life which require precise regulation. The bone remodeling consists of bone formation performed by osteoblasts and bone resorption performed by osteoclasts. The balance between these processes maintains bone homeostasis and bone renewal. However, in pathophysiological conditions including osteoporosis, diabetes, obesity, aging and rare bone diseases, bone remodeling is impaired causing poor bone quality and higher risk of fractures. Therefore, it is important to understand the molecular mechanism regulating bone cell differentiation in health and disease. This Special Issue focused on the topic of osteoblast and osteoclast differentiation in bone physiology in context of different bone diseases. It collected several original research articles presenting novel findings regarding the regulation of bone homeostasis.

## Impact of various bioactive molecules on osteoclastogenesis and osteogenesis

The study by Zhu et al., found that peiminine, an alkaloid obtained from the bulb of *Fritillaria thunbergii Miq* prevented osteoclast differentiation and function. *In vitro* treatment with peiminine reduced the number of osteoclast cells in a dose dependent manner and suppressed fusion of osteoclast cells *via* inhibition of M-CSF and GST-rRANKL and decreased activation of NF-κB. Further, peiminine treament in ovariectomized mice (OVX) slowed down bone resorption and prevented bone loss. Thus, this study showed that peiminine affects osteoclast function, which makes it a potential candidate molecule to prevent osteoporosis.

Next, Yu et al., investigated whether glucagon-like peptide-1 receptor (GLP-IR) agonists such as liraglutide could prevent bone loss in type 1 diabetes (T1D). Using female streptozotocin-induced diabetic C57BL/6J mice, liraglutide treatment for 8 weeks improved glucose metabolism in comparison to placebo treatment, which was accompanied by improved trabecular and cortical bone parameters. Further, molecular analysis using RNA-sequencing showed that liraglutide treatment in T1D mice decreased osteoclastogenesis and inflammation. Thus, this study demonstrates that liraglutide prevents T1D-induced bone fagility by suppression of osteoclastogenesis.

Further, Zheng et al. explored the effect of melatonin on osteogenesis and angiogenesis in osteoporotic fracture healing. In this study, bilateral ovariectomy was done in rat model followed by monocortical tibia defect. The animals were treated with melatonin for 4 weeks, resulting in improvement of bone healing and bone strength compared to control group. Further, immunohistochemical staining indicated higher expression levels of osteogenesis-related marker (OCN) and angiogenesis-related markers (VEGF and CD31) in the melatonin-treated OVX rats. Thus, these findings suggest a positive effect of melatonin on fracture healing in osteoporotic condition.

The study by Goodnough et al., compared the response of mouse and human SSCs to Non-Steroidal Anti-Inflammatory Drugs (NSAIDs) in fracture healing, as NSAIDs are used in the early inflammatory responses during bone fracture. Using primary SSCs from fractures the authors found that the mouse SSCs treated with NSAIDs were impaired in osteochondrogenic differentiation, while human SSCs were not affected. These findings point out the cross-species differences in response to NSAID and its sensitivity during fracture healing.

## Different angles on signaling pathways involved in osteogenesis

The bone remodeling is an active dynamic process by coordinating action of bone resident osteoblasts and osteoclasts, which is dysregulated in diseases with high bone turnover rates and transforming growth factor beta 1 (TGF-β1). The study by Chen et al. reported the role of TGF-β1 signaling in bone resorption in patients with Camurati-Engelmann disease (CED). This study reported a higher level of active Rho GTPases and migration-related proteins Integrin β1and β3 in peripheral blood in this disease patients. TGF-β1 activates Rho to increase osteoclast formation and bone resorption, with simulatenous enhancment of migration of pre-osteoclasts and mature osteoclasts. Whereas hyperactive TGF-β1-stimulated osteoclastogenesis was rescued by inhibition of Rho GTPases *in vitro.* Thus, these data assume that crosstalk between Rho and TGF-β1 leads to increase bone turnover in CED. A complex multifactorial disorder osteoporosis is associated with various risk factors and medical conditions, with bone marrow-derived mesenchymal stem cell dysfunction being a crutial factor. The study by Li et al. identified upregulation of miR-4739 in BMSC of osteoporotic subjects. Further, cell viability, osteoblast differentiation, and heterotopic bone formation were diminished by overexpression of miR-4739, whereas miR-4739 inhibition showed opposite effects. Thus, this study presented a novel signaling pathway regulating osteoblast differentiation in osteoporosis.

The study by Cheng et al. identified a role of neural cell adhesion molecule (NCAM) in osteoblast differentation, Downregulation of NCAM in MC3T3-E1 resulted in decreased expression of osteogenic markers *RunX2* and *Osterix*, and decreased deposition of calcium, hence diminishing osteoblast differentiation. Similarly β-catenin levels and Akt phosphorylation levels were reduced. Thus these findings assumed that NCAM plays a pivotal role in the regulation of signaling pathways involved in osteoblast differentiation.

Gu et al. determined the role of super-enhancer-associated LINC01485/miR-619-5p/RUNX2 signaling in osteogenic differentiation. During osteogenic induction expression level of LINC01485 was increased along with osteogenic genes including RUNX2, and OCN. Over-expression of LINC01485 *in vitro* induced up-regulation of osteogenic genes and ALP activity, while knockdown caused an opposite effect on osteoblast differentiation. The RAP assay identified miR-619-5p as a candidate binding partner. Further data showed that LINC01485 competes with miR-619-5p to promote expression of RUNX2. Thus, this study points out that osteogenesis is promoted by LINC01485 whose activation is controled by miR-619-5p.

## Link between bone resident cells and diabetes

Obesity and diabetes impair bone remodeling and slow down bone turnover causing increased risk of fractures. In their study, Ballato et al. investigated the presence of circulating osteoblast progenitors (COP) and circulating osteoclast progenitors (OCP) in relation to type 2 diabetes (T2D) and bone turnover. This clinical study reported that patients with poor glycemic control had higher percentage of COP in the circulation comparison to healthy controls suggesting a copying mechanism to increased osteoblast apoptosis in the hyperglycemic condition in diabetes. The authors suggested that high COP could be a marker of a poor glycemic control and predictor of disturbances of bone homeostasis.

Further study by Phimphilai et al. reported that T2D patients showed impaired osteoblast differentiation in peripheral blood derived mononuclear cells (PBMC) due to increased expression of receptor of advance glycation end products (RAGE). They found that younger age of T2D patients had a protective effect on osteoblast differentiation, while chronic exposure to T2D caused higher RAGE activation in PMBC of T2D patients associated with impaired osteoblast differentiation. These patients had increased BAX/BCL2 ratio, which was negatively correlated with osteoblast markers. Thus, these data suggest that, RAGE activation and age contributed to the diminished osteogenic differentiation potential of PBMC in T2D patients.

In fact, this especial issue provides new insights on the bone cell differentiation in different conditions and experimental settings. We believe that this Issue opens new questions which will be followed in future by basic and translational studies.

## Author contributions

All authors listed have made a substantial, direct, and intellectual contribution to the work and approved it for publication.

